# Supplementary material for: VIGA: a one-stop tool for eukaryotic virus identification and genome assembly from next-generation-sequencing data
Source: Brief Bioinform. 2023 Dec 2;25(1):bbad444. doi: 10.1093/bib/bbad444 (PMC10753531; doi:10.1093/bib/bbad444)
Supplement: TableS1_S4_bbad444 [file tables1_s4_bbad444.docx]

**Supplementary Tables**

**Table S1.** Performance of different assembly tools on the HIV dataset. ^#^Normalized with the method of Min-Max Normalization.

| Method | Strain Precision (%) | Normalized Strain Precision# | Genome Fraction (%) | Normalized Genome Fraction# | Mismatches per 100 kbp | Normalized Mismatches per 100 kbp# |
| --- | --- | --- | --- | --- | --- | --- |
| VIGA | 100 | 1.00 | 98.20 | 1.00 | 2787.00 | 0.93 |
| MetaCompass | 50 | 0.18 | 60.45 | 0.00 | 1463.00 | 0.96 |
| VirGenA | 100 | 1.00 | 76.14 | 0.42 | 2766.80 | 0.93 |
| Trinity | 39 | 0.00 | 97.19 | 0.97 | 39585.60 | 0.00 |
| Haploflow | 100 | 1.00 | 93.36 | 0.87 | 33.70 | 1.00 |

**Table S2.** Performance of different assembly tools on the HBV dataset. ^#^Normalized with the method of Min-Max Normalization.

| Method | Strain Precision (%) | Normalized Strain Precision# | Genome Fraction (%) | Normalized Genome Fraction# | Mismatches per 100 kbp | Normalized Mismatches per 100 kbp# |
| --- | --- | --- | --- | --- | --- | --- |
| VIGA | 100 | 1.00 | 99.91 | 1.00 | 1890.65 | 1.00 |
| MetaCompass | 100 | 1.00 | 99.91 | 1.00 | 2397.20 | 0.64 |
| VirGenA | 100 | 1.00 | 46.12 | 0.00 | 1942.18 | 0.96 |
| Trinity | 74 | 0.00 | 73.48 | 0.51 | 3306.42 | 0.00 |
| Haploflow | 100 | 1.00 | 91.19 | 0.84 | 2355.00 | 0.67 |

**Table S3**. The performance of VIGA and its competitor tools on assembling virus genomes for HBV and HHV5. The accession numbers of the relevant NGS data in NCBI SRA database were ERR3253398- ERR3253399 and SRR5629574, respectively, while the accession numbers of virus genomes in NCBI GenBank database were MK720628.1, MK720631.1 and EF999921.1, respectively. ^*^, only viral reads were input for genome assembly as VirGenA cannot deal with large datasets. ^#^ Strain precision is defined as the ratio of correctly assembled contigs among all contigs of the virus.

| Method | HBV (3212-3215 bp; GC: 48.74%) | | | HHV5 (229050 bp; GC: 57.36%) | | |
| --- | --- | --- | --- | --- | --- | --- |
|  | Genome fraction(%) | Mismatches per 100 kbp | Strain precision# | Genome fraction(%) | Mismatches per 100 kbp | Strain precision# |
| VIGA | 99.91 | 1890.65 | 100% (4/4) | 99.47 | 25.46 | 100% (1/1) |
| MetaCompass | 99.91 | 2397.20 | 100% (4/4) | 99.26 | 25.51 | 100% (3/3) |
| Haploflow | 91.19 | 2355.00 | 100% (13/13) | 34.50 | 1.27 | 0.6% (51/8594) |
| Trinity | 73.48 | 3306.42 | 73.8% (31/42) | 99.08 | 81.52 | 0.6% (188/30988) |
| VirGenA | 46.12 | 1942.18 | 100% (5/5) | 98.41* | 5.32* | 100% (6/6*) |

**Table S4**. The performance of VIGA and its competitor tools on assembling virus genomes for Suid herpesvirus 1 strain Kaplan (SuHV-1) and Diachasmimorpha longicaudata entomopoxvirus (DlEPV). The accession numbers for the relevant NGS data in NCBI SRA database were ERR908011 and SRR8167468, respectively, while the accession numbers of virus genomes in NCBI GenBank database were KJ717942.1 and KR095315.1 respectively. *, only viral reads were input for genome assembly as VirGenA cannot deal with large datasets. # Strain precision is defined as the ratio of correctly assembled contigs among all contigs of the virus.

| Method | SuHV-1 (143423 bp; GC: 73.59%) | | | DlEPV (252940 bp; GC: 30.07%) | | |
| --- | --- | --- | --- | --- | --- | --- |
|  | Genome fraction(%) | Mismatches per 100 kbp | Strain precision# | Genome fraction(%) | Mismatches per 100 kbp | Strain precision# |
| VIGA | 85.48 | 680.28 | 100% (2/2) | 92.56 | 589.87 | 100% (2/2) |
| MetaCompass | 78.15 | 632.53 | 100% (67/67) | 91.74 | 537.80 | 100% (49/49) |
| Haploflow | 1.05 | 0.00 | 100% (2/2) | 24.95 | 280.51 | 0.8% (71/8374) |
| Trinity | 68.10 | 58.36 | 99.5% (370/372) | 75.08 | 1159.57 | 0.9% (203/22343) |
| VirGenA* | 18.06 | 77.21 | 100% (37/37) | 70.57 | 287.97 | 100% (99/99) |
